# Low red/far-red ratio can induce cytokinin degradation resulting in the inhibition of tillering in wheat (*Triticum aestivum* L.)

**DOI:** 10.3389/fpls.2022.971003

**Published:** 2022-12-08

**Authors:** Kangqi Lei, Qingwen Tan, Liqi Zhu, Libing Xu, Shuke Yang, Jinling Hu, Lijun Gao, Pan Hou, Yuhang Shao, Dong Jiang, Weixing Cao, Tingbo Dai, Zhongwei Tian

**Affiliations:** ^1^ Key Laboratory of Crop Physiology Ecology and Production Management of Ministry of Agriculture, Nanjing Agricultural University, Nanjing, Jiangsu, China; ^2^ National Agricultural Exhibition Center (China Agricultural Museum), Chaoyang District, Beijing, China

**Keywords:** wheat, tillering, low R/FR, hormone, cytokinin, degradation

## Abstract

Shoot branching is inhibited by a low red/far-red ratio (R/FR). Prior studies have shown that the R/FR suppressed *Arabidopsis thaliana* branching by promotes bud abscisic acid (ABA) accumulation directly. Given that wheat tiller buds are wrapped in leaf sheaths and may not respond rapidly to a R/FR, systemic cytokinin (CTK) may be more critical. Here, systemic hormonal signals including indole-3-acetic acid (IAA), gibberellins (GA) and CTK and bud ABA signals in wheat were tested under a low R/FR. The results showed that a low R/FR reduced the percentage of tiller occurrence of tiller IV and the tiller number per plant. The low R/FR did not rapidly induced ABA accumulation in the tiller IV because of the protection of the leaf sheath and had little effect on IAA content and signaling in the tiller nodes. The significant change in the CTK levels was observed earlier than those of other hormone (ABA, IAA and GA) and exogenous cytokinin restored the CTK levels and tiller number per plant under low R/FR conditions. Further analysis revealed that the decrease in cytokinin levels was mainly associated with upregulation of cytokinin degradation genes (*TaCKX5*, *TaCKX11*) in tiller nodes. In addition, exposure to a decreased R/FR upregulated the expression of GA biosynthesis genes (*TaGA20ox1*, *TaGA3ox2*), resulting in elevated GA levels, which might further promote CTK degradation in tiller nodes and inhibit tillering. Therefore, our results provide evidence that the enhancement of cytokinin degradation is a novel mechanism underlying the wheat tillering response to a low R/FR.

## Introduction

Tillering of wheat (*Triticum aestivum* L.) is an important determinant of spike density and grain production. The appropriate tiller number and spike density in wheat can be obtained by changing the cultivation measures such as planting density. A higher planting density results in earlier tillering cessation and fewer tillers per plant, which is related to the red/far-red ratio (R/FR) to a certain extent (Evers et al., 2006; Sparkes et al., 2006). In plant populations, red light is absorbed by the surrounding canopy, whereas most far-red light is transmitted and reflected, causing the decrease in the R/FR. Increased planting density results in a faster decline in the R/FR (Sparkes et al., 2006). A high R/FR increases, but a low R/FR decreases the total tiller number (Ugarte et al., 2010). Cessation of tiller bud outgrowth starts at a relatively conservative R/FR of 0.2-0.4 (Evers et al., 2006; Sparkes et al., 2006; Dreccer et al., 2013). However, few studies have reported the physiological mechanisms of a low R/FR on tillering in wheat, which limits cultivation and genetic improvement for regulating the tillering response to a low R/FR and planting density in wheat.

A low R/FR is detected by the phytochromes family, including the phytochrome B (phyB) which is the key receptor (Sawers et al., 2005; Casal, 2013). A low R/FR inactivated phyB, and elongation-promoting PHYTOCHROME INTERACTING FACTORs (PIFs) are released to regulate downstream genes expression (Leivar and Monte, 2014). In Arabidopsis (*Arabidopsis thaliana*), phyB deficiency and a low R/FR have been shown to inhibit shoot branching by regulating hormone signals (Finlayson et al., 2010; Reddy et al., 2013; Yao and Finlayson, 2015). Several studies have stated that abscisic acid (ABA) is the key signal regulates Arabidopsis axillary bud outgrowth responses to the R/FR (Reddy et al., 2013; Yao and Finlayson, 2015; Holalu and Finlayson, 2017). ABA abundance in buds rapidly decreased in response to a high R/FR before Arabidopsis axillary bud growth changes (Holalu and Finlayson, 2017). Under a decreased R/FR, elevated ABA was related to upregulated biosynthesis gene expression (Reddy et al., 2013) and was found to downregulate the expression of cell cycle-related genes to inhibit Arabidopsis axillary bud outgrowth (Yao and Finlayson, 2015). Importantly, Arabidopsis axillary buds exposed to air can directly perceive a low R/FR; however, wheat tiller buds before they grew out, may not directly perceive a low R/FR due to the protection of leaf sheaths. In other words, wheat tiller buds could not produce ABA signals as quickly, so ABA probably is not the key hormone mediates the inhibiting effect of a low R/FR on wheat tillering.

Systemic hormonal signals from the main stem such as indole-3-acetic acid (IAA), gibberellins (GA) and cytokinin (CTK) are involved in the regulation of shoot branching (Wang et al., 2018) and may be more critical for the regulation of wheat tillering by the R/FR. IAA synthesized at the shoot apex is well known for its role in apical dominance, but it does not enter the bud directly from the main stem (Cline and Choonseok, 2006). One model proposes that IAA can reduce CTK levels in nodes by downregulating the expression of biosynthesis genes and therefore inhibit branching (Tanaka et al., 2006). Active GA, such as GA_1_ and GA_4_, inhibit tiller bud outgrowth. GA levels in nodes are determined by biosynthesis, including the actions of GA 20-oxidase (GA20ox) and GA 3-oxidase (GA3ox), and degradation including the action of GA 2-oxidase (GA2ox) (Binenbaum et al., 2018). GA-deficient mutants exhibit higher shoot branching than wild types and reduced GA levels produce increased branching phenotypes in rice (Lo et al., 2008). GA-inhibition of tillering is mainly due to decreased CTK levels by upregulating CTK degradation genes by GA (Zhuang et al., 2019), indicating that CTK could act as the key messenger. CTK from roots and basal nodes can directly move into the bud to promote outgrowth (Barbier et al., 2019). The *trans*-zeatin (*t*Z) and *trans*-zeatin riboside (*t*ZR; transport form of *t*Z) are natural cytokinin that are mainly synthesized by the actions of phosphate-isopentenyltransferase (IPT) and cytochrome P450 monooxygenases (CYP735As), degraded by cytokinin oxidase/dehydrogenase (CKX), conjugated by cytokinin glucosyltransferase (CGT), or reverted by glucosidase (GLU) (Chen et al., 2021). An intuitively clear mechanism for CTK-mediated bud outgrowth is promoting the cell cycle by inducing the expression of cell-cycle-related genes (Müller and Leyser, 2011).

Previous studies have shown that a low R/FR promotes the IAA content in young *Arabidopsis* seedlings (Tao et al., 2008; Hornitschek et al., 2012), and systemic auxin signaling is necessary for maintained *Arabidopsis* bud suppression by a decreased R/FR (Holalu et al., 2021). Two studies have reported that the GA biosynthetic genes *GA20ox* and *GA3ox* in *Rumex palustris* (Pierik et al., 2011) and *KAO* in *Pinus tabuliformis* (Li et al., 2020) are upregulated by a low R/FR. A study on sorghum (*Sorghum bicolor*) suggested that the dormant state of *phyB-1* buds is correlated with the regulation of cytokinin degradation genes (Kebrom and Mullet, 2016). Furthermore, PIFs was found to directly induce *AtCKX5* expression based on ChIP-seq results (Leivar and Monte, 2014). Since both IAA and GA inhibit shoot branching by reducing CTK content in nodes, we addressed the question whether CTK abundance and degradation were the key factors mediate the inhibiting effect of a low R/FR on wheat tillering. It was hypothesized that a low R/FR would alter CTK homeostasis by inducing degradation genes expression, thus inhibiting wheat tillering. To prove our hypotheses, we monitored the temporal patterns of tiller number per plant, tiller bud outgrowth and hormonal state under a low R/FR. And we used two techniques to characterize hormonal state, involving the measurement of hormone levels and gene expression.

## Materials and methods

### Materials and experimental design

A pot experiment was conducted at the Pailou Experimental Station of NJAU, Nanjing, China (32°04′N, 118°76′E) during the 2020–2021 growing season. The concentration of organic matter, total nitrogen, available phosphorus and available potassium in the sieved soil were 19.84 g kg^-1^, 0.76 g kg^-1^, 28.24 mg kg^-1^, and 86.42 mg kg^-1^, respectively. Each pot (length 49.5 cm, width 21 cm, height 20 cm) was filled with 16 kg of sieved soil and placed outdoors. Each pot was supplied with 10.5 g of compound fertilizer (15% nitrogen, 15% phosphorus, and 15% potassium) before sowing, and the other 1.0 g nitrogen was top-dressed at the jointing stage. Yangmai 25 (YM25, *Triticum aestivum* L.), one of the popular wheat varieties in Jiangsu province, was selected to be sown in the drill and thinned to 14 seedlings with a seedling spacing of 3.2 cm per pot at the three-leaf stage, and coleoptile tillers were removed simultaneously. Two most marginal seedlings in each pot were not sampled to reduce the marginal effect.

Two treatments were designed: natural radiation supplemented with far-red light (Low R/FR) and a control (Control). The experimental setting and measurement of the R/FR were performed as described by Ugarte et al. (2010) with minor modifications, as shown in **Supplementary Figure S1**. A 40 W LED plant light provided far-red light (15 μmol m^-2^ s^-1^, 730 ± 10 nm). Each lamp was east-west oriented, 50 cm away from the pots, placed at plant height, and the plants were horizontally irradiated from the south side from 8 am to 5 pm daily from the five-leaf stage to the booting stage. The whole plant was exposed to FR light, and the red/far-red ratio at the base of the plant was characterized by an SKR 116 dual-channel radiometer (Skye Instruments, Llandrindod Wells, UK). The red/far-red ratio was calculated as the average of the ratio measured facing towards the light sources and the ratio measured facing against the light sources. Low R/FR treatment reduced the red/far-red ratio received by the plants from 0.78 (Control) to 0.38 at noon on a sunny day.

Wheat tiller dynamics and plant height (two pots per replicate, three biological replicates) were observed every 7 days after treatment (DAT), and phenotypes were recorded at the booting stage. The tiller buds exposed from the leaf sheath and became new tillers were counted, excluding dying tillers whose newest leaves started yellowing. The percentage of tiller occurrence (%) for the tiller position was equal to the tiller number of the tiller position/total number of plants (%). Considered that the percentage of tillers I, II and III from the main stem was 100% at five-leaf stage, the tiller IV (from the axil of the fourth leaf on the main stem) was studied. For morphology, hormone levels, and gene expression analysis, the tiller node and tiller IV were harvested with three biological replicates at 7, 14, and 21 DAT. The seedlings were dug out and washed, and then the fourth leaf (from bottom) and its leaf sheath were peeled off. The tiller IV and its corresponding tiller node were cut respectively. All fresh samples were immediately placed in liquid N_2_ and then stored at -80°C. Samples were dried at 105 °C for 30 min and then at 75 °C for 72 h.

To verify that a low R/FR inhibited tillering by reducing CTK levels in wheat, a pharmacology experiment was conducted to study the alleviating effect of 6-benzyl aminopurine (synthetic cytokinin; 6-BA) on the suppressed tillering of wheat under low R/FR conditions in October 2021. Given that the *t*Z-type cytokinin were synthesized from the roots and basal nodes, 6-BA was applied to the roots using a hydroponic experiment. YM25 was planted in the Hoagland’s nutrient solution, containing 0.75 mM Ca(NO_3_)_2_, 1 mM KNO_3_, 1 mM KH_2_PO_4_, 0.5 mM CaCl_2_, 0.5 mM CaSO_4_, 1 mM MgSO_4_, 10 mM Fe-EDTA, 0.5 mM NaCl, 2.35 mM H_3_BO_3_, 0.0385 mM ZnSO_4_7H_2_O, 0.55 mM MnSO_4_H_2_O, 0.0165 mM CuSO_4_5H_2_O and 0.0065 mM H_2_MoO_4_, following the detailed hydroponic methods from Gao et al. (2018). The Control and Low R/FR treatments were remained the same as in the pot experiment, and 6-BA (25 μM) was added to the solutions at 7 DAT under low R/FR conditions (Low R/FR + 6-BA). The red/far-red ratios in the Control, Low R/FR and Low R/FR + 6-BA treatments were 0.75, 0.35, and 0.34, respectively, at noon on 14 DAT. Tiller number and tiller IV growth of wheat treated for 7 days and 14 days and the control, as well as the CTK levels in tiller nodes at 7 and 14 DAT and the control were measured.

### Hormone levels assays

Plant hormone (ABA, IAA, GA_1_, GA_4_, *t*Z, and *t*ZR) levels were measured according to Kojima et al. (2009) with minor modifications. Fresh powder samples (0.2 g) was homogenized in 5 mL pre-chilled acetonitrile for 12 h. The supernatant was collected by centrifugation at 12,000 x *g* for 15 min at 4°C. The pellet was resuspended in 5 mL pre-chilled acetonitrile for 2 h and then collected and combined with the supernatant after centrifugation as before. Next, 20 mg C18 powder and 0.5 g anhydrous magnesium sulfate powder was added to the combined supernatant, and the supernatant was collected after centrifugation as before. The supernatant was concentrated to near dryness using a centrifuge concentrator for 6–8 h and added with 0.2 mL methanol. The sample was filtered through a 0.22 μm organic nylon membrane and filled with a 2 mL sample bottle. The levels of plant hormones were measured using liquid chromatography-mass spectrometry (LC-MS/MS; QTRAP^®^ 6500, AB Sciex, USA) fitted with a chromatographic column (ACQUITY UPLC HSS T3, 1.8 μm, 2.1 × 75 mm, Waters). Hormones were separated at a flow rate of 0.3 mL min^-1^ with the gradients of solvent A (0.1% formic acid) and solvent B (acetonitrile) set as follows: 0 min, 95% A + 5% B; 1 min, 95% A + 5% B; 8 min, 5% A + 95% B; 8.1 min, 95% A + 5% B; and 10 min, 95% A + 5% B.

### RNA extraction and cDNA synthesis

Total RNA was extracted from tiller node and bud tissues using TRIzol reagent (Vazyme Bio, China) according to the manufacturer’s protocols. cDNA synthesis was performed using HiScript III Q RT SuperMix (Vazyme Bio, China), following the manufacturer’s instructions. The cDNA samples were diluted 5× before qPCR analysis.

### Primers

To ensure the reliability of the results, two or three typical genes with high expression in each metabolic hormone process were tested in tiller nodes. The primer sequences for a part of target genes including *TaNCED*, *TaABA3*, *TaCYP707A1*, *TaCYP707A2*, *TaIAA12*, *TaIAA13*, *TaARF13*, *TaARF15*, *TaGA20ox1*, *TaGA20ox2*, *TaGA3ox2*, *TaGA2ox1*, *TaGA2ox4*, *TaGA2ox6*, *TaGLU6*, *TaGLU7*, *TaGLU13*, and *TaADP-RF* (reference gene) have been reported previously (Paolacci et al., 2009; Pearce et al., 2015; Son et al., 2016; Izydorczyk et al., 2018; Qiao et al., 2018; Shoaib et al., 2019; Lang et al., 2021). The primer sequences for other target genes were designed using the Primer3 software (https://bioinfo.ut.ee/primer3-0.4.0/) and are listed in **Supplementary Table S1**. The amplification efficiency of the total genes ranged from 90 to 105%. The sequences of some of these genes, including *TaCYP735A1*, and *TaCYP735A2*, have not yet been reported in wheat. Thus, the CDS sequences of Arabidopsis *AtCYP735A1* (GenBank ID: AT5G38450), and *AtCYP735A2* (GenBank ID: CYP735A2) published in NCBI were used for a BLAST search against the Chinese Spring gene (RefSeq v1.0 or v2.1) in WheatOmics 1.0 (Ma et al., 2021). Gene sequences with the highest similarity were selected, and the corresponding primers were previously described and are listed in **Supplementary Table S1**.

### Real-time qPCR assay

qPCR was performed using the CFX Connect Real-Time PCR Detection System (Bio-Rad, USA) with ChamQ SYBR qPCR Master Mix (Vazyme Bio, China). The PCR conditions involved a preliminary incubation for 30 s at 95 °C, followed by 40 cycles of 10 s at 95°C, and 30 s at 58 or 60°C. Relative transcript levels were calculated using the method described by Livak and Schmittgen (2001) with *TaADP-RF* as the reference gene. Three biological replicates and two technical replicates were performed for each gene.

### Statistical analysis

Statistically significant differences between the control and treated samples were tested using Student’s *t*-test (*P* < 0.05) using SPSS software (version 22.0, SPSS Inc., Chicago, IL, USA).

## Results

### Morphology trait

**Figure 1:**
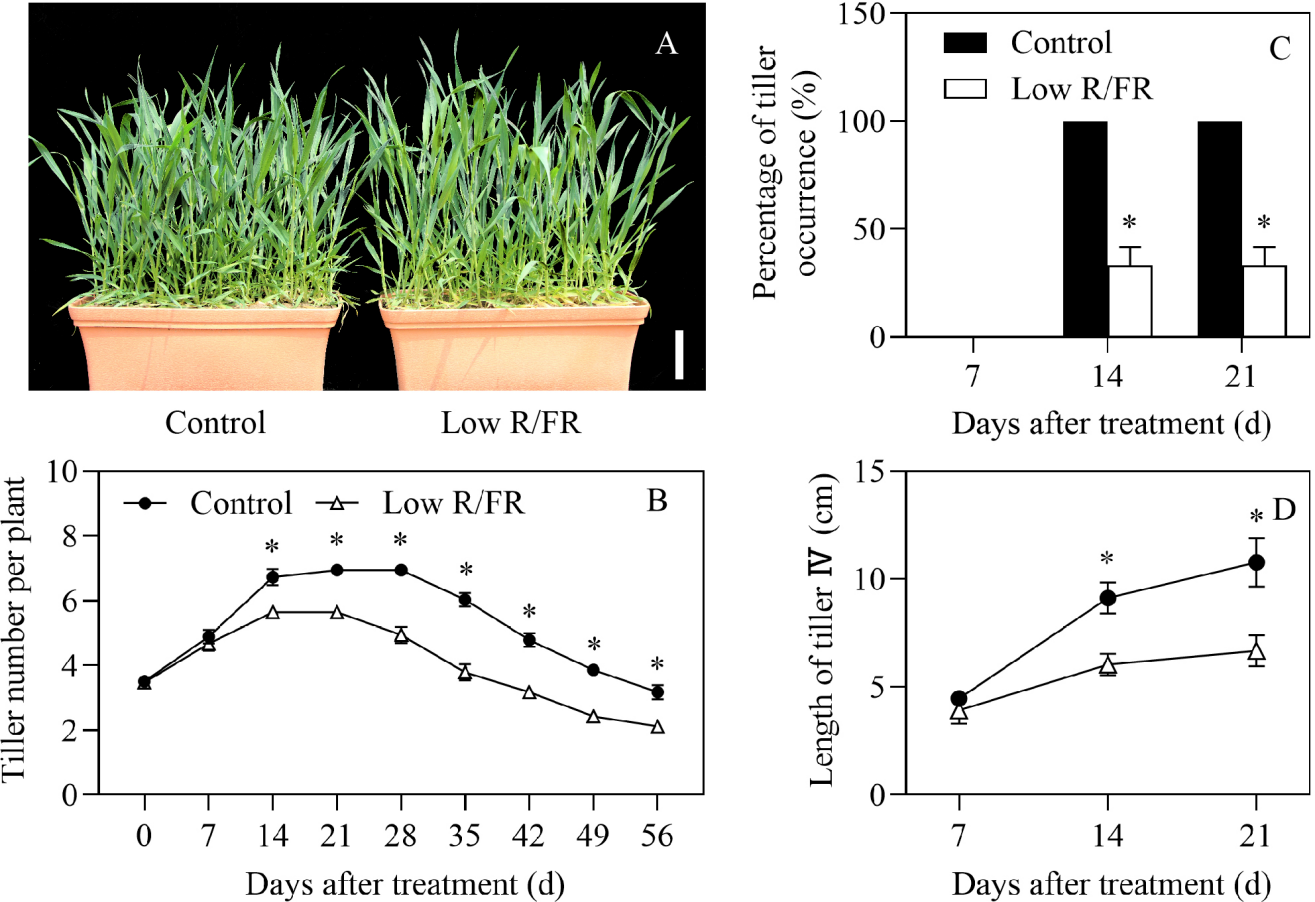
Plants and tillers phenotype under control and low R/FR conditions. **(A)** Comparison of phenotype at booting stage (56 DAT), and the white legend represents 10 cm. **(B)** Tiller number per plant was tested at 0, 7, 14, 21, 28, 35, 42, 49 and 56 DAT. **(C)** The percentage of tiller occurrence of tiller IV was counted at 7, 14 and 21 DAT. Tiller IV means the tiller bud from the axil of the 4^th^ leaf from bottom on the main stem. **(D)** Length of tiller IV was determined at 7, 14 and 21 DAT. Data are the means of three biological replicates ± SE, and the asterisk denotes a statistically remarkable difference (*P* < 0.05) between treated and control samples. DAT, days after treatment.

Exposure to a low R/FR resulted in a 16% decrease in tiller number per plant at 14 DAT and the effect lasted until the end of the treatment compared to the control (**Figures 1A, B**). Due to a decreased R/FR, tillering stopped at 14 DAT and declined at 28 DAT, which was earlier than that of the control at 21 DAT and 35 DAT, respectively (**Figure 1B**). The percentage of tiller occurrence of the tiller IV was zero at 7 DAT and significantly reduced by 67% at 14 and 21 DAT under low R/FR conditions (**Figure 1C**), indicating that tiller IV buds were wrapped in leaf sheaths and did not become new tillers at 7 DAT and tiller IV growth was inhibited by a low R/FR at 14 and 21 DAT. The tiller IV buds were not significantly different in size at 7 DAT and the length of tiller IV was reduced at 14 and 21 DAT under low R/FR conditions (**Figure 1D**). These results indicated that wheat tillering was inhibited significantly in response to a low R/FR.

### Hormone levels

**Figure 2:**
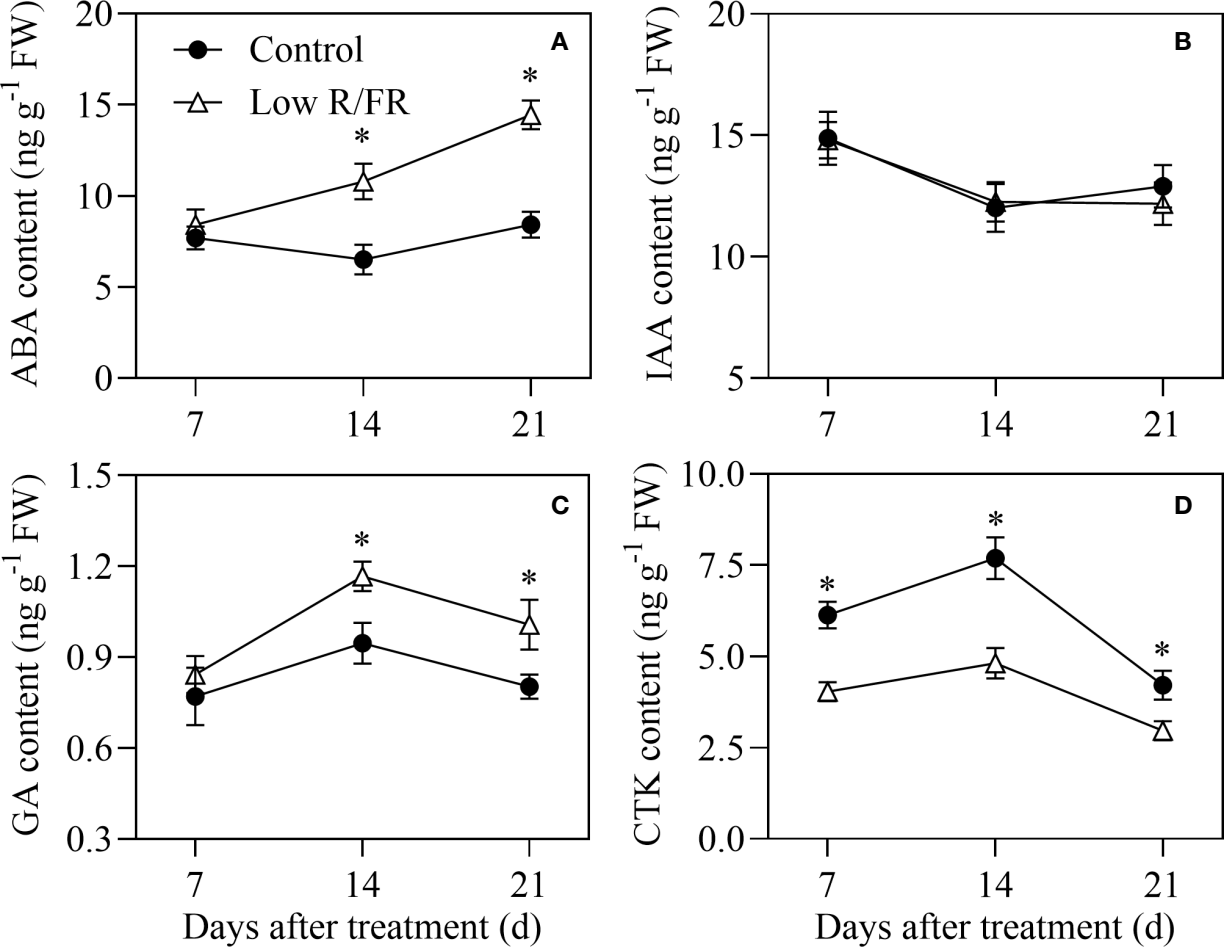
Abscisic acid (ABA) abundance in the tiller IV and auxin, gibberellin (GA) and cytokinin (CTK) levels in tiller nodes and under control and low R/FR conditions. ABA **(A)** levels in the tiller IV and indole-3-acetic acid (IAA;) **(B)**, GA **(C)** and CTK **(D)** levels in tiller nodes treated for 7, 14 and 21 d. GA levels were the sum of GA_1_ and GA_4_. CTK levels were the sum of *t*Z and *t*ZR. Data are the means of three biological replicates ± SE, and the asterisk denotes a statistically remarkable difference (*P* < 0.05) between treated and control samples.

There was no difference in ABA levels in the tiller IV at 7 DAT, and ABA levels were significantly higher in the tiller IV at 14 and 21 DAT (66–72%) by a low R/FR compared to the control (**Figure 2A**). These results indicated that a decreased R/FR did not rapidly induced ABA accumulation in the tiller IV wrapped in leaf sheaths until the tiller IV grew out. Tiller nodes IAA levels were not affected by a low R/FR at any DAT (**Figure 2B**), but GA levels were significantly increased at 14 and 21 DAT (23–25%) under low R/FR conditions relative to the control (**Figure 2C**). Tiller nodes CTK levels were decreased by 34% at 7 DAT and this effect was pronounced as the treatment continued for 14 d and 21 d (**Figure 2D**). Overall, CTK abundance in tiller nodes declined in response to a decreased R/FR prior to measured effects on tiller growth and other hormone levels.

### Transcriptional regulation of ABA metabolism in the tiller IV

**Figure 3:**
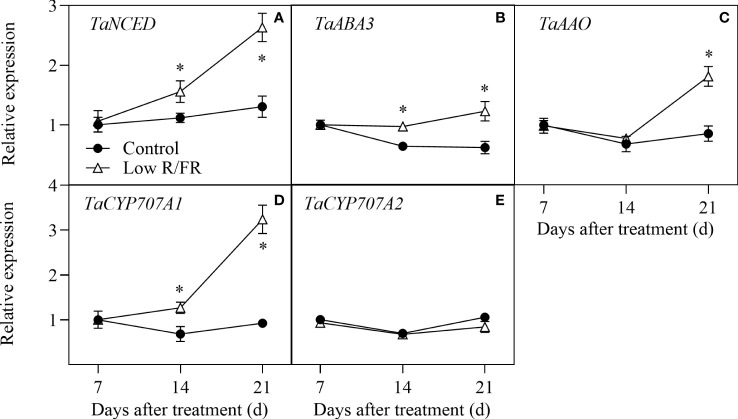
Expression of ABA metabolism genes in the tiller IV under control and low R/FR conditions. Relative transcript levels of *TaNCED* **(A)**, *TaABA3* **(B)**, *TaAAO* **(C)**, *TaCYP707A1* **(D)**, and *TaCYP707A2* **(E)** in the tiller IV treated for 7, 14 and 21 d. Data are the means of three biological replicates ± SE, and the asterisk denotes a statistically remarkable difference (*P* < 0.05) between treated and control samples.

The relative expression levels of ABA biosynthesis (*TaNCED*, *TaABA3*, and *TaAAO*) and deconjugation (*TaCYP707A1* and *TaCYP707A1*) genes in tiller nodes at all DATs were showed in **Figure 3**. No significant effect of a low R/FR on the transcripts of above genes was observed at 7 DAT (**Figures 3A–E**), further suggesting that a decreased R/FR did not rapidly induced ABA accumulation in the tiller IV. Compared to the control, the relative expression levels of *TaNCED*, *TaABA3*, and *TaCYP707A1* were upregulated at 14 and 21 DAT by a low R/FR (**Figures 3A, B, D**). In addition, the relative expression levels of *TaAAO* were improved at 21 DAT and *TaCYP707A1* was remained unchanged at all DATs under low R/FR conditions (**Figures 3C, E**). Thus, a low R/FR improved ABA levels by induced ABA biosynthesis genes (*TaNCED* and *TaABA3*) in the tiller IV.

### Transcriptional regulation of auxin signaling in tiller nodes

**Figure 4:**
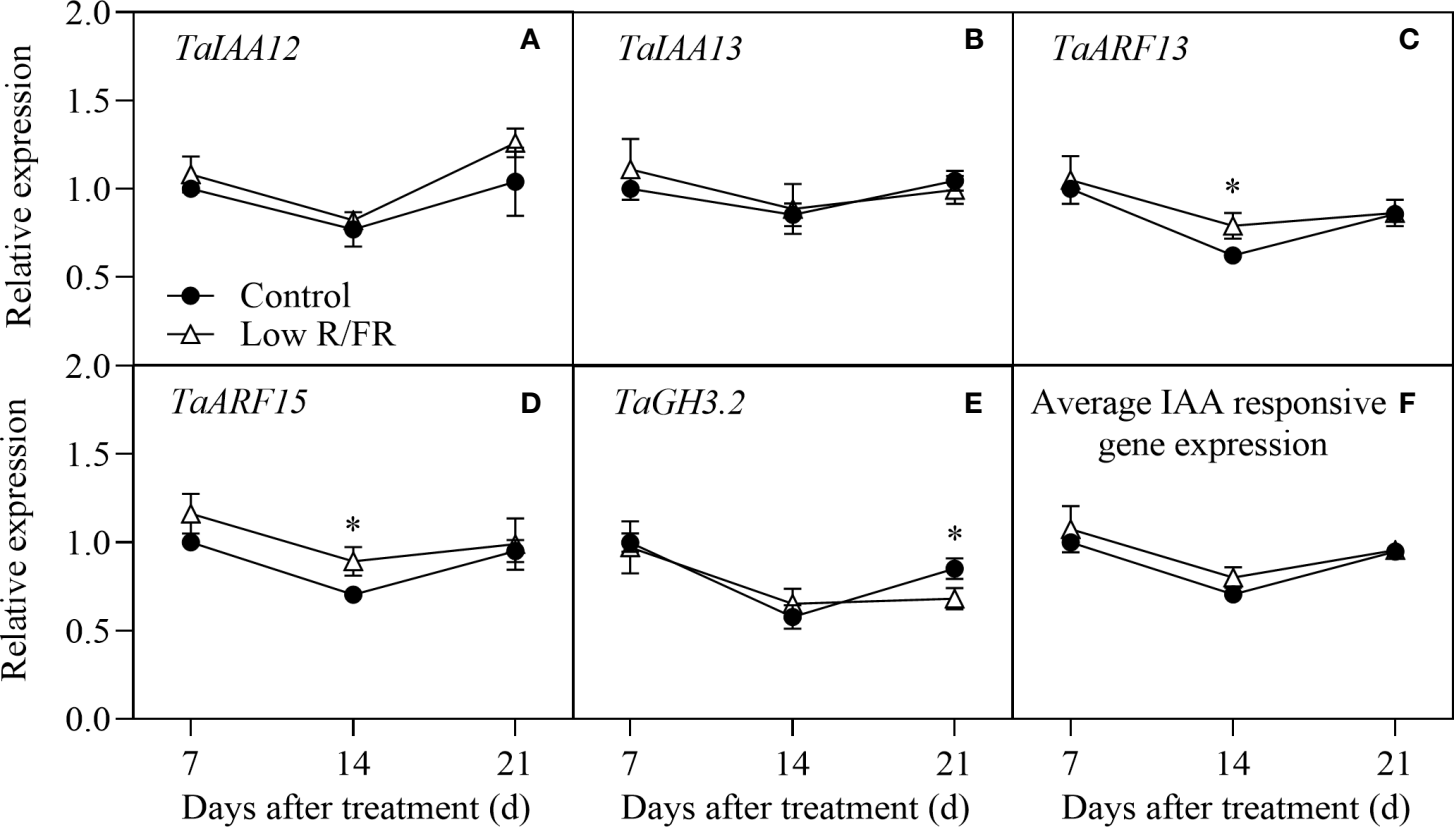
Expression of IAA-responsive genes in tiller nodes under control and low R/FR conditions. Relative transcript levels of *TaIAA12* **(A)**, *TaIAA13* **(B)**, *TaARF13* **(C)**, *TaARF15* **(D)**, *TaGH3.2* **(E)**, and average positive normalized expression **(F)** in tiller nodes treated for 7, 14 and 21 d. Data are the means of three biological replicates ± SE, and the asterisk denotes a statistically remarkable difference (*P* < 0.05) between treated and control samples.

Auxin signaling status was assessed using a panel of IAA-responsive genes, including *TaIAA12*, *TaIAA13*, *TaARF13*, *TaARF15*, and *TaGH3.2* in tiller nodes at all DATs. No significant effect of a low R/FR on the transcripts of *TaIAA12* and *TaIAA13* was observed at all DATs (**Figures 4A, B**). Relative to the control, the expression of *TaARF13* and *TaARF15* was upregulated at 14 d after treatment, but there were no differences at any other days (**Figures 4C, D**). In addition, the relative expression level of *TaGH3.2* was decreased at 21DAT to a lesser extent by a low R/FR (**Figure 4E**). The average relative expression of the auxin signaling genes was not altered by the R/FR (**Figure 4F**). In summary, a low R/FR had little effect on auxin signaling in tiller nodes.

### Transcriptional regulation of GA metabolism in tiller nodes

**Figure 5:**
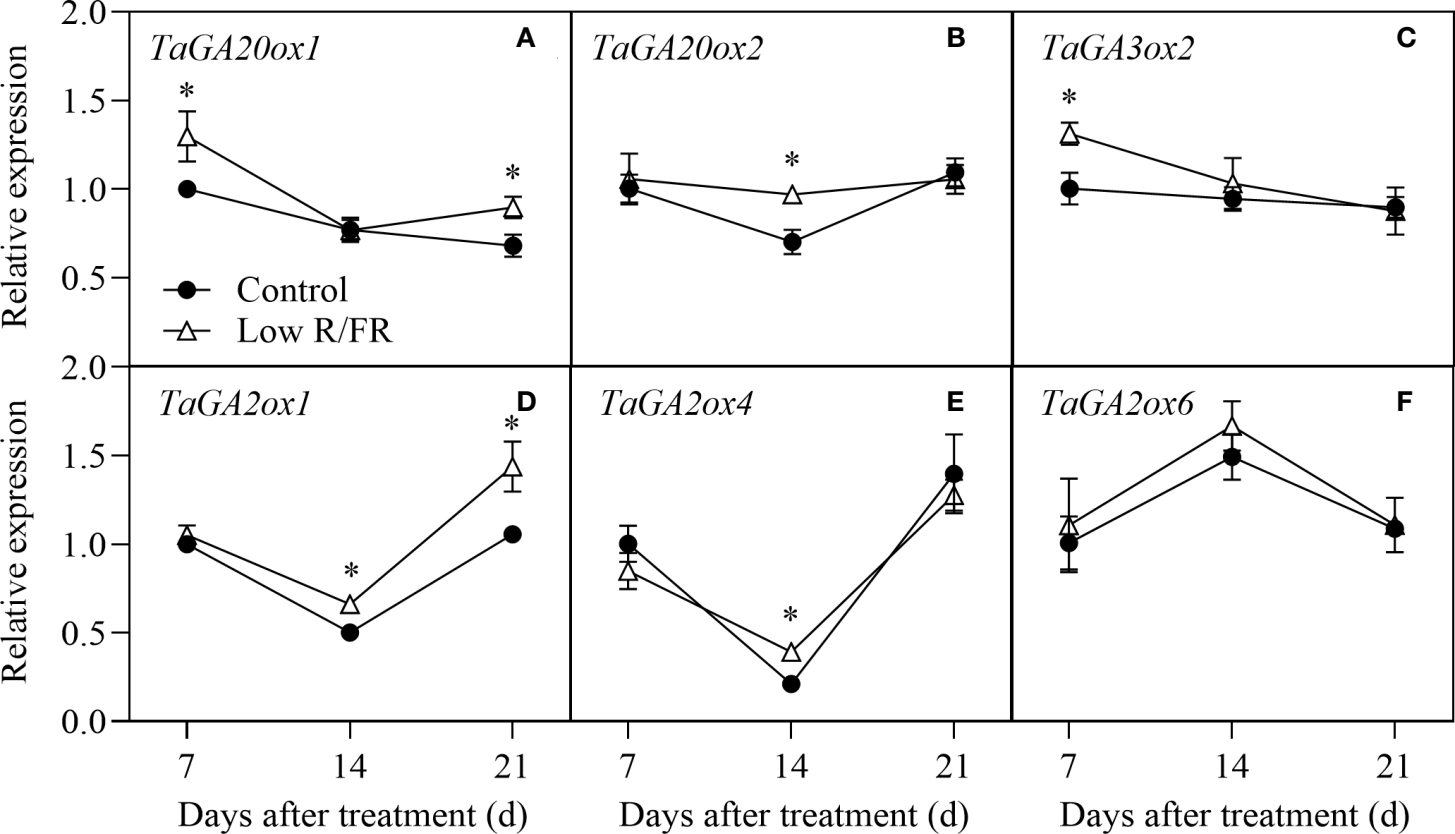
Expression of GA metabolism genes in tiller nodes under control and low R/FR conditions. Relative transcript levels of *TaGA20ox1* **(A)**, *TaGA20ox2* **(B)**, *TaGA3ox2* **(C)**, *TaGA0ox1* **(D)**, *TaGA2ox4* **(E)**, and *TaGA2ox6* **(F)** in tiller nodes treated for 7, 14 and 21 d. Data are the means of three biological replicates ± SE, and the asterisk denotes a statistically remarkable difference (*P* < 0.05) between treated and control samples.

The relative expression levels of GA biosynthesis (*TaGA20ox1*, *TaGA20ox2*, and *TaGA3ox2*) and degradation (*TaGA2ox1*, *TaGA2ox4*, and *TaGA2ox6*) genes at all DATs were showed in **Figure 5**. The relative expression levels of the GA biosynthetic genes *TaGA20ox1* and *TaGA3ox2* significantly increased at 7 DAT and that of *TaGA20ox2* significantly increased at 14 DAT in response to a low R/FR (**Figures 5A–C**). Among the three degradative genes, the relative expression levels of *TaGA2ox1* and *TaGA2ox4* were significantly upregulated at 14 DAT, whereas *TaGA2ox6* was not affected by a low R/FR (**Figures 5D–F**). These results suggested that the increase in GA levels was associated with upregulated biosynthesis genes (*TaGA20ox1* and *TaGA3ox2*) in wheat under low R/FR conditions.

### Transcriptional regulation of CTK metabolism in tiller nodes

**Figure 6:**
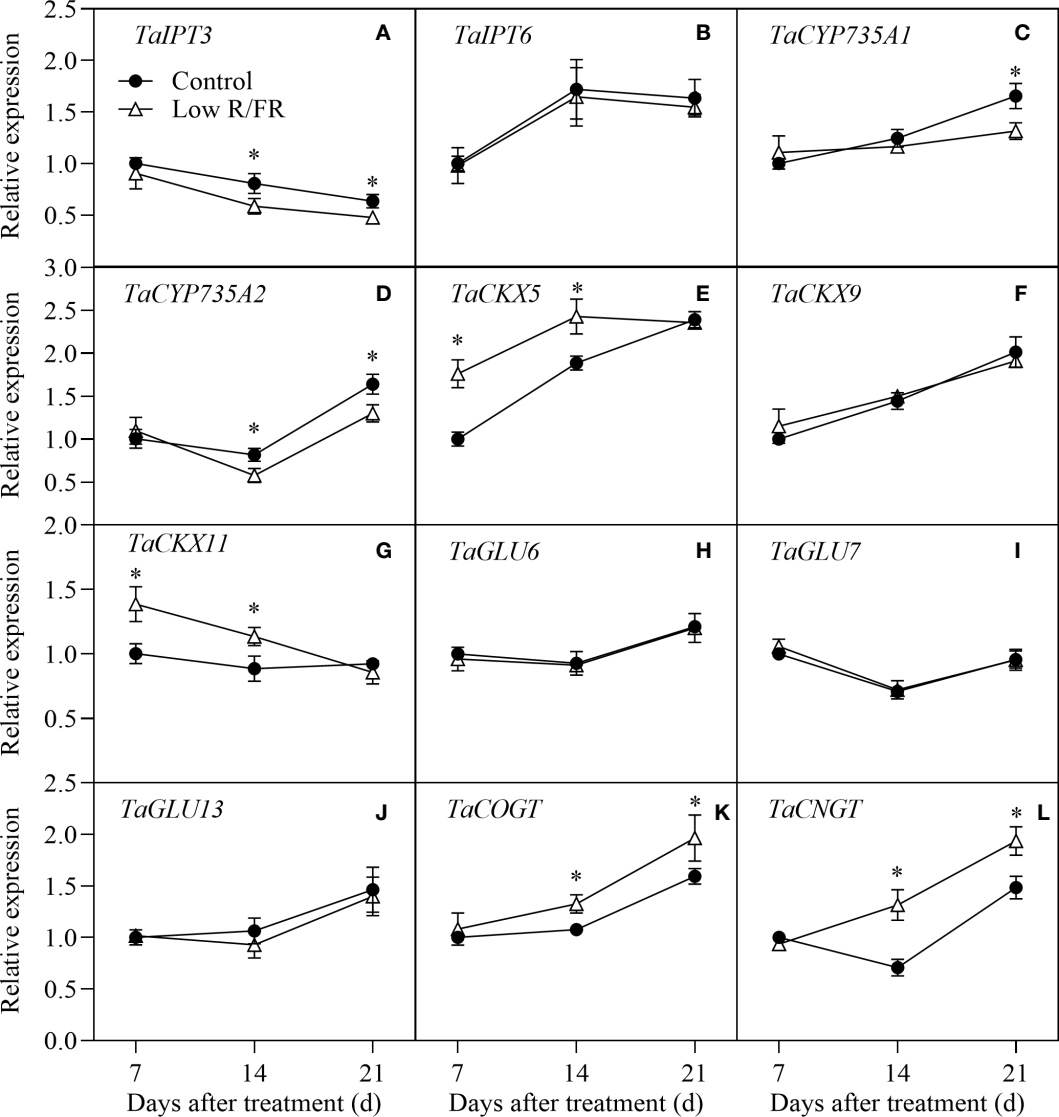
Expression of CTK metabolism genes in tiller nodes under control and low R/FR conditions. Relative transcript levels of *TaIPT3* **(A)**, *TaIPT6* **(B)**, *TaCYP735A1* **(C)**, *TaCYP735A2* **(D)**, *TaCKX5* **(E)**, *TaCKX9* **(F)**, *TaCKX11* **(G)**, *TaGLU6* **(H)**, *TaGLU7* **(I)**, *TaGLU13* **(J)**, *TaCOGT* **(K)** and *TaCNGT* **(L)** in tiller nodes treated for 7, 14 and 21 D. Data are the means of three biological replicates ± SE, and the asterisk denotes a statistically remarkable difference (*P* < 0.05) between treated and control samples.

The relative expression patterns of CTK biosynthesis (*TaIPT3*, *TaIPT6*, *TaCYP735A1*, and *TaCYP735A2*), degradation (*TaCKX5*, *TaCKX9*, and *TaCKX11*), deconjugation (*TaGLU6*, *TaGLU7*, and *TaGLU13*) and conjugation (*TaCOGT* and *TaCNGT*) genes in tiller nodes at all DATs were showed in **Figure 6**. Treatment with a low R/FR downregulated the relative expression levels of *TaIPT3* and *TaCYP735A2* at 14 and 21 DAT and the expression of *TaCYP735A1* at 21 DAT; however, it did not affect the expression of *TaIPT6* in tiller nodes (**Figures 6A–D**). The relative expression levels of *TaCKX5* and *TaCKX11* were higher in low R/FR conditions than that in the control conditions at 7 and 14 DAT, and the expression of *TaCKX9* was unchanged under low R/FR conditions (**Figures 6E–G**). Low R/FR treatment did not alter the relative expression levels of *TaGLU6*, *TaGLU7* and *TaGLU13* in tiller nodes (**Figures 6H–J**). In addition, Low R/FR treatment resulted in a marked increase in the relative expression levels of *TaCOGT* and *TaCNGT* in tiller nodes at 14 and 21 DAT compared with the Control (**Figures 6K, L**). Overall, the decrease in CTK levels was associated with inhibited biosynthesis genes (*TaIPT3*, *TaCYP735A2*) and enhanced degradation (*TaCKX5*, *TaCKX11*) and conjugation (*TaCOGT*, *TaCNGT*) genes in wheat under low R/FR conditions.

### Effect of 6-BA on tiller number and length under low R/FR conditions

**Figure 7:**
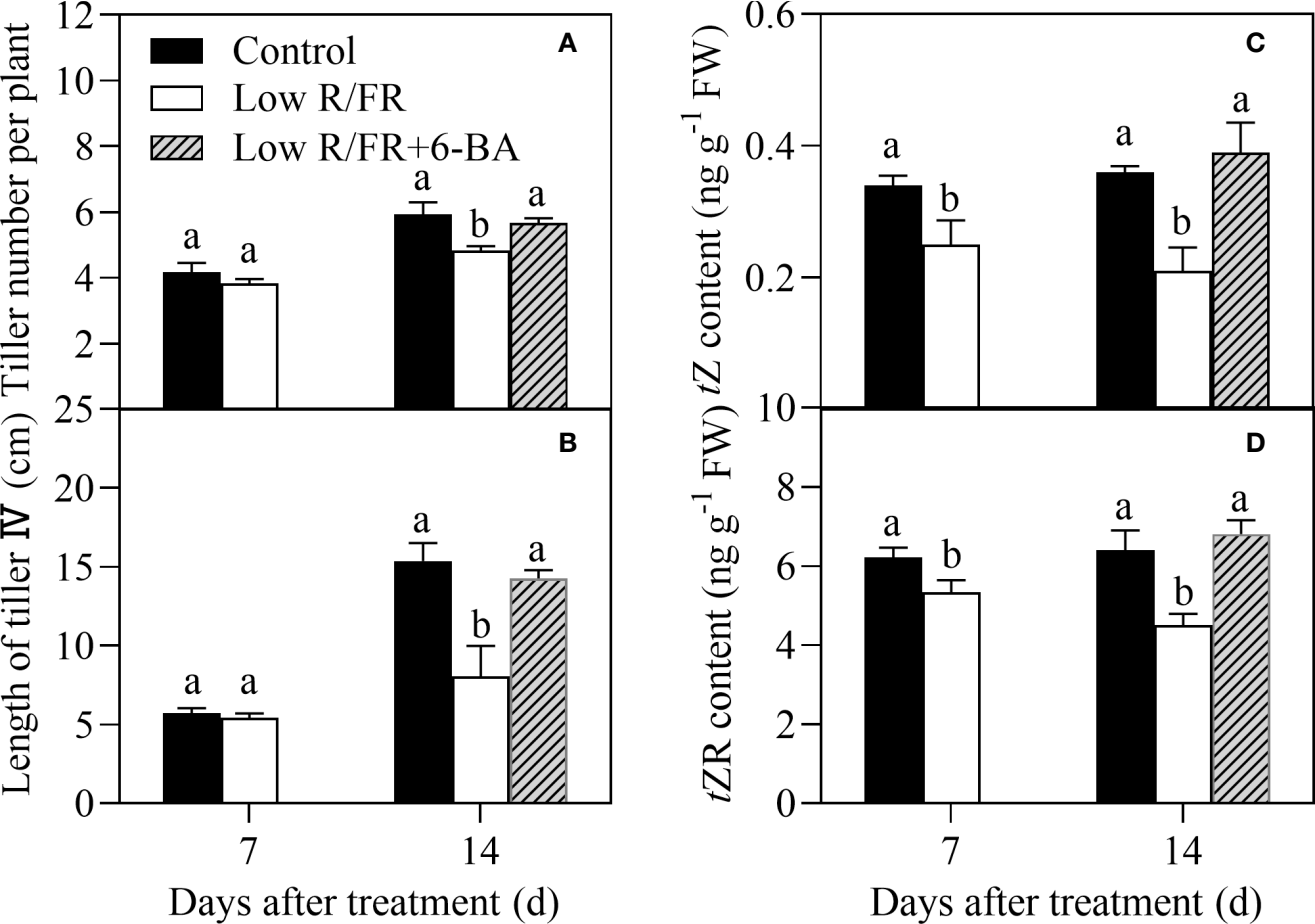
Effect of root application 6-BA on tiller number and tiller IV growth under low R/FR conditions (hydroponic experiment). Tiller numbers per plant **(A)**, length of tiller IV **(B)** and levels of *t*Z **(C)** and *t*ZR **(D)** in tiller nodes were tested at 7, 14 DAT. Data are the means of three biological replicates ± SE, and the lowercase letters denotes a statistically remarkable difference (*P* < 0.05) between treated and control samples.

Similarly, exposure to a low R/FR resulted in a marked decrease in tiller number per plant, length of tiller IV, and levels of *t*Z and *t*ZR at 14 DAT in the hydroponic experiment (**Figure 7**). Importantly, root application of 6-BA (exogenous cytokinin) treatment significantly improved tiller number per plant, length of the tiller IV, and levels of *t*Z and *t*ZR under low R/FR conditions (**Figure 7**). These results suggested that root-supplied 6-BA increased the levels of *t*Z-type CTK in tiller nodes and improved tillering under low R/FR conditions.

## Discussion

A low R/FR triggers shade avoidance syndrome, characterized by a decrease in branch number (Fernández-Milmanda and Ballaré, 2021). In this study, a low R/FR reduced tiller number and caused earlier tillering cessation and decline (**Figures 1A, B**). Similar effects of a decreased R/FR have also been reported in wheat (Ugarte et al., 2010; Xie et al., 2016). Further analysis revealed that the reduction in tiller number was associated with a lower percentage of tiller occurrence of tiller IV under low R/FR conditions (**Figure 1C**). The regulation of branching by a low R/FR is a complex process. Endogenous hormones, including IAA, GA, ABA, and CTK, are involved in the regulation of bud outgrowth (Wang et al., 2018). The present results showed CTK levels in tiller nodes declined in response to a low R/FR prior to measured effects on tiller growth and other hormone levels (**Figure 1**, **2**). And the application of exogenous cytokinin (6-BA) in roots recovered endogenous CTK levels and tiller number (**Figure 7**), suggesting that cytokinin could be the key hormone mediating the effect of the R/FR on tiller bud outgrowth in wheat. Increase in endogenous CTK levels in the tiller nodes in response to the 6-BA application is in accordance with the reports available (Xu et al., 2015; Yang et al., 2020), and probably due to the promotion of cytokinin biosynthesis by the 6-BA application (Panda et al., 2018; He et al., 2020).

Initial work indicated that rapid responses to an increased R/FR might be mediated by changes in bud ABA in *Arabidopsis thaliana* (Reddy et al., 2013; Yao and Finlayson, 2015; Holalu and Finlayson, 2017). However, a low R/FR did not rapidly induce ABA accumulation in the tiller IV (**Figure 2A**), because the wheat tiller IV bud was wrapped by the leaf sheath within 7 DAT in our study (**Figure 1C**). Moreover, no significant effect of a decreased R/FR on the relative expression of ABA mechanism genes was observed at 7 DAT (**Figures 3**). These results confirmed our assumption that wheat tiller buds, unlike Arabidopsis axillary buds, respond weakly to a low R/FR before they were exposed from the leaf sheath. Thus, ABA was not the key hormone that inhibited wheat tillering by a low R/FR. Nonetheless, the effect of ABA could not be completely ignored, as a large accumulation of ABA was observed in the tiller IV at 14 and 21 DAT under low R/FR conditions (**Figure 2A**). Accumulation of ABA was attributed to upregulation of biosynthesis genes (*TaNCED* and *TaABA3*) by a decreased R/FR in our study, and these results are consistent with previous findings (Reddy et al., 2013). ABA promotes plant senescence (Zhang and Gan, 2012) and a low R/FR may initiate tiller death in wheat (Sparkes et al., 2006). In our study, elevated ABA levels may exacerbate tiller death, explaining that a low R/FR causes earlier decline in tiller number.

Auxin and GA were not the key hormones that mediated the regulation of a low R/FR in our study. Prior studies have shown that the indole-3-acetic acid (IAA) accumulation or signaling increases rapidly in young Arabidopsis seedlings under a low R/FR (Tao et al., 2008; Hornitschek et al., 2012; Reddy and Finlayson, 2014). However, IAA levels and expression levels of IAA signaling genes in tiller nodes were not altered by a low R/FR in this study (**Figures 2B**, **4**). This could be because IAA mainly mediates the increase in plant height rather than the decrease in bud outgrowth response to a low R/FR (Tao et al., 2008). These results are in agreement with those of Holalu and Finlayson (2017), showing that the auxin content and signaling in the main stem presented a weak and transient response to low R/FR. However, GA may still be involved in inhibiting wheat tillering. These findings showed that the increase in GA levels was associated with improved biosynthesis in wheat (**Figures 2C**, **5**), as suggested by Pierik et al. (2011) for *Rumex palustris*. The upregulation of gibberellin degradation might maintain gibberellin homeostasis under a low R/FR. Given that GA-inhibition of tillering was related to decreased CTK levels by enhancing CTK degradation in nodes (Zhuang et al., 2019), GA was most likely involved in promoting CTK degradation and inhibiting wheat tillering in our study.

Cytokinin levels are related to biosynthesis, degradation, conjugation, and deconjugation (Chen et al., 2021). The relative expression levels of CTK biosynthesis genes (*TaIPT3* and *TaCYP735A2*) were downregulated by a low R/FR following 14 DAT (**Figures 6A, D**). This could be explained by the fact that these two genes are specifically induced by nitrate (Sakakibara et al., 2006) which was reduced in our study (data not shown). The relative expression levels of conjugation genes (*TaCOGT*, *TaCNGT*) were improved by a low R/FR at 14 and 21 DAT (**Figures 6K, L**), suggesting that enhanced conjugation by a low R/FR might contribute to lower levels of CTK. Cytokinin glucosyltransferase is thought to function in the overabundance or overproduction of CTK (Hoyerová and Hošek, 2020). Our results provide insights into the role of CGT in cytokinin homeostasis. The relative expression levels of degradation genes (*TaCKX5*, *TaCKX11*) were notably upregulated by a low R/FR at 7 DAT prior to other genes (**Figures 6E**,G), indicating the reduction in CTK levels was mainly related to enhanced degradation. A previous study reported that CTK levels in *phyB-1* buds could be lower than those in wild-type buds due to increased expression of the cytokinin degradation gene (*SbCKX1*) (Kebrom and Mullet, 2016), suggesting that our results are credible. In other words, a low R/FR can induce cytokinin degradation resulting in the inhibition of wheat tillering. What needs to be considered is that the tiller node is not an organ that directly senses the R/FR, so there should be the other substance to transmit the signal. Prior work have shown that PIFs may directly improve *AtCKX5* expression (Leivar and Monte, 2014). We speculated that PIFs would be shoot-to-root mobile signals which induce *TaCKXs* expression in the tiller nodes. Linking environmental control of branching with specific genes may provide a method to alter crop architecture (Doust, 2007). It is feasible to use gene editing technology such as CRISPR for inhibiting the expression of *TaCKXs* to regulate the sensitivity of wheat tillering to a low R/FR. For this, the relationship between a low R/FR and *TaCKXs* in wheat should be further verified using molecular genetics. It is important to note that increasing the tiller number in high-density populations would increase the risk of lodging. Therefore, this improvement method is suitable for varieties sensitive to a low R/FR and planting density.

Sucrose has been shown to play a signaling role in bud outgrowth (Mason et al., 2014; Wang et al., 2018). It is interesting to explore that whether sucrose is involved in a low R/FR regulation of wheat tillering and the interaction between sucrose and cytokinin in the control of tillering by the R/FR. The other limitation of the present study is a lack of research on strigolactone (SL), which is involved in bud outgrowth regulation by a low R/FR or shade (Kebrom et al., 2010; Yao and Finlayson, 2015). Because the difficulty in determining the SL level and the limited understanding of its metabolic process in biosynthesis, this study did not conduct in-depth research on the role of SL in regulating wheat tillering response to a low R/FR. Given that SL is mainly synthesized from roots, the inhibiting effect may need more time to manifest. Although SL signaling genes in axillary buds of *Dendranthema grandiflorum* were rapidly induced by a low R/FR (Yuan et al., 2018), this induction would be probably prevented by the presence of leaf sheaths in wheat. Therefore, physiological mechanisms in response to a low R/FR are different for plants with different branching structures, so that species characteristics need to be considered when modifying the sensitivity to a low R/FR.

## Conclusion

This study provides evidence that a low R/FR can decrease CTK levels in tiller nodes by inducing degradation genes expression, thus inhibiting wheat tillering. The low R/FR did not rapidly induce ABA accumulation in the tiller bud because the bud was wrapped by the leaf sheath. Although the node IAA content and signaling were not altered under low R/FR conditions, the elevated GA levels might further promote CTK degradation in tiller nodes and inhibit tillering. Further studies will explore the relationship between a low R/FR and cytokinin degradation at the molecular level.

## Data availability statement

The original contributions presented in the study are included in the article/supplementary material, further inquiries can be directed to the corresponding author/s.

## Author contributions

KL, ZT and TD conceived and designed the research. KL, LX and LZ conducted experiments. SY and JH contributed to data collection. QT, LG and PH contributed to data analysis. KL wrote the manuscript. DJ and WC supported the resources, QT, YS, TD and ZT revised the manuscript. All authors contributed to the article and approved the submitted version.
