# Supplementary material for: Low red/far-red ratio can induce cytokinin degradation resulting in the inhibition of tillering in wheat (Triticum aestivum L.)
Source: Front Plant Sci. 2022 Dec 8;13:971003. doi: 10.3389/fpls.2022.971003 (PMC9773260; doi:10.3389/fpls.2022.971003)
Supplement: Supplementary file 1 [file DataSheet_1.docx]

Supplementary Material


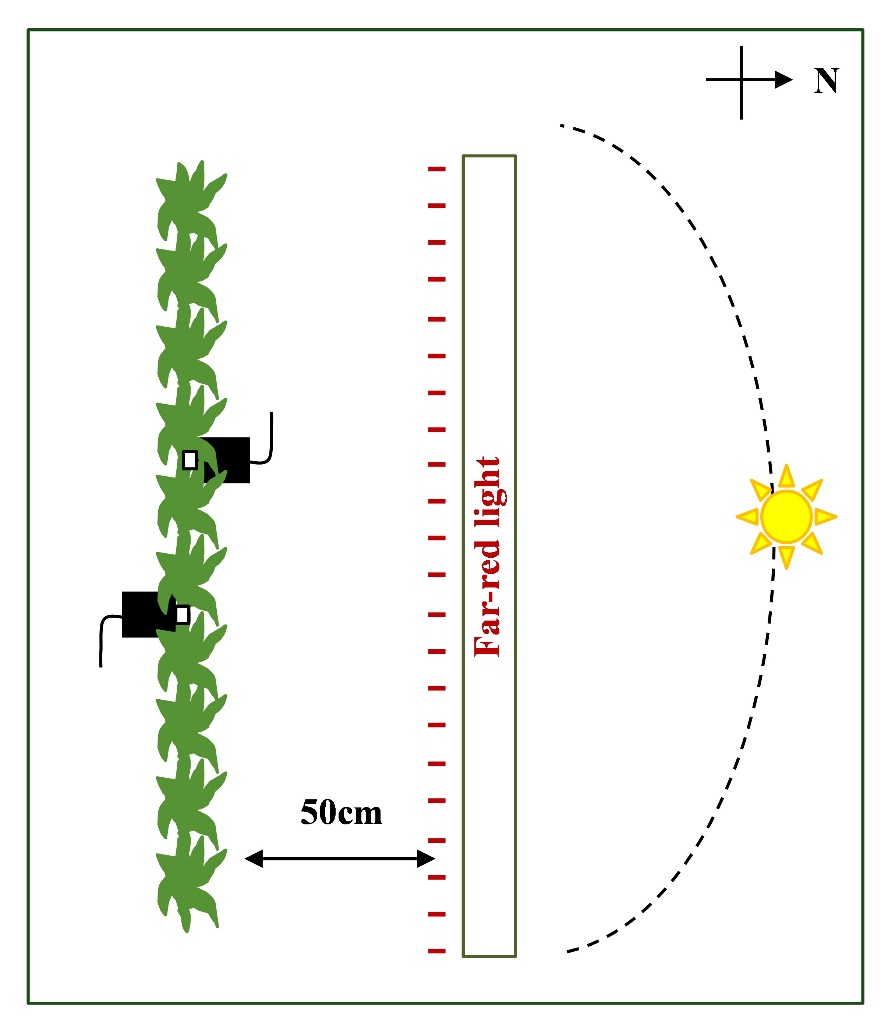


**Supplementary Figure 1.** Model diagram of far-red light apparatus and determination of R/FR. Each lamp was east-west oriented, 50 cm apart from the pots, placed at plant height, and horizontally irradiated from the south. The R/FR ratio was equal to the average of the ratio measured facing towards the far-red light and the ratio measured facing against the far-red light.

**Supplementary Table 1.** Gene ID, primers sequence and amplicon length for target genes.

| Gene name | IWGSC Sequence ID | Primer sequence (5’ → 3’) | | Amplicon length (bp) |
| --- | --- | --- | --- | --- |
| *TaAAO* | TraesCS5A02G427800.1 | F: | GCCCAAGCGAACATGGCAAG | 176 |
|  | TraesCS5B02G429800.1 | R: | AGGCTCTGCCCACAGTCGTA |  |
|  | TraesCS5D02G435900.1 |  |  |  |
| *TaGH3.2* | TraesCS1A02G425100.1 | F: | TGTAGCTGCGAGGAGACCAT | 236 |
|  | TraesCS1B02G459500.1 | R: | ACTGGTGCGCTATCACCATC |  |
|  | TraesCS1D02G434100.1 |  |  |  |
| *TaIPT3* | TraesCS3A01G263300 | F: | GGGTCCAACTCCCTCATCCA | 85 |
|  | TraesCS3B01G296500 | R: | ATCGACCCAGAGGAGACAGC |  |
|  | TraesCS3D01G263000 |  |  |  |
| *TaIPT6* | TraesCS5A01G460000 | F: | AAGCACAAGGCCGTCGTC | 99 |
|  | TraesCS5B01G469600 | R: | CTGCATCTTGTCGGAGTTGA |  |
|  | TraesCS5D01G471100 |  |  |  |
| *TaCYP735A1* | TraesCS6A02G012100.1 | F: | CGTCTCTACCCTCCGGTCGT | 177 |
|  | TraesCS6B02G018500.2 | R: | GCCCTGCTCGAACCTCATGG |  |
|  | TraesCS6D02G015000.1 |  |  |  |
| *TaCYP735A2* | TraesCS5A02G204300.1 | F: | GAGGGCGTCGTCATGGAGTC | 165 |
|  | TraesCS5B02G203900.1 | R: | CCGCGTGTCGTAGCTGAACT |  |
|  | TraesCS5D02G211800.1 |  |  |  |
| *TaCKX5* | TraesCS3A01G321100 | F: | CACAAGTCGCAGCCCGAGTG | 95 |
|  | TraesCS3B01G344600 | R: | AGCATGGCCCTCGGATCGAA |  |
|  | TraesCS3D01G310200 |  |  |  |
| *TaCKX9* | TraesCS1A01G234800 | F: | GCCTCAGGTGGAGAACTCTG | 175 |
|  | TraesCS1B01G248700 | R: | CCAGTTCGTTCACATTGCTG |  |
|  | TraesCS1D01G237200 |  |  |  |
| *TaCKX11* | TraesCS7A01G536900 | F: | CGCCTGCAGATGAAGCTCGT | 146 |
|  | TraesCS7B01G455000 | R: | GTGAGCCGGAGGTAGTCCGT |  |
|  | TraesCSUn01G106300 |  |  |  |
| *TaCOGT* | TraesCS3A01G125500 | F: | GGTGAGGAGGTCCAGAGCCA | 159 |
|  | TraesCS3B01G144600 | R: | GGCAGGGTCATCGAGCTTCC |  |
| *TaCNGT* | TraesCS3A02G466200 | F: | GCTCCGGCGTGACCTCGATA | 156 |
|  | TraesCS3B01G510100 | R: | AGGCTGCCGAAGCTCACGTA |  |
|  | TraesCS3D01G461200 |  |  |  |

Note: F, forward primer; R, reverse primer.
